# Extracellular Vesicles in NAFLD/ALD: From Pathobiology to Therapy

**DOI:** 10.3390/cells9040817

**Published:** 2020-03-27

**Authors:** Alejandra Hernández, Juan Pablo Arab, Daniela Reyes, Ainhoa Lapitz, Han Moshage, Jesús M. Bañales, Marco Arrese

**Affiliations:** 1 Departamento de Gastroenterologia, Escuela de Medicina, Pontificia Universidad Catolica de Chile. Santiago, Chile 8330077; ale.hernandez.vill@gmail.com (A.H.); jparab@gmail.com (J.P.A.); dbreyes@uc.cl (D.R.); 2 Department of Gastroenterology and Hepatology, University of Groningen, University Medical Center Groningen, 9713 GZ Groningen, The Netherlands; a.j.moshage@umcg.nl; 3 Centro de Envejecimiento y Regeneracion (CARE), Departamento de Biología Celular y Molecular, Facultad de Ciencias Biológicas Pontificia Universidad Catolica de Chile, Santiago 8331010, Chile; 4 Biodonostia Health Research Institute, Donostia University Hospital, 20014 San Sebastian, Spain; ainhoa.lapitz@biodonostia.org (A.L.); JESUS.BANALES@biodonostia.org (J.M.B.); 5 National Institute for the Study of Liver and Gastrointestinal Diseases (CIBERehd), Carlos III National Institute of Health, 28029 Madrid, Spain; 6 IKERBASQUE, Basque Foundation for Science, 48013 Bilbao, Spain

**Keywords:** nonalcoholic fatty liver disease, fatty liver, alcoholic liver disease, extracellular vesicles, signaling, exosomes, biomarkers

## Abstract

In recent years, knowledge on the biology and pathobiology of extracellular vesicles (EVs) has exploded. EVs are submicron membrane-bound structures secreted from different cell types containing a wide variety of bioactive molecules (e.g., proteins, lipids, and nucleic acids (coding and non-coding RNA) and mitochondrial DNA). EVs have important functions in cell-to-cell communication and are found in a wide variety of tissues and body fluids. Better delineation of EV structures and advances in the isolation and characterization of their cargo have allowed the diagnostic and therapeutic implications of these particles to be explored. In the field of liver diseases, EVs are emerging as key players in the pathogenesis of both nonalcoholic liver disease (NAFLD) and alcoholic liver disease (ALD), the most prevalent liver diseases worldwide, and their complications, including development of hepatocellular carcinoma. In these diseases, stressed/damaged hepatocytes release large quantities of EVs that contribute to the occurrence of inflammation, fibrogenesis, and angiogenesis, which are key pathobiological processes in liver disease progression. Moreover, the specific molecular signatures of released EVs in biofluids have allowed EVs to be considered as promising candidates to serve as disease biomarkers. Additionally, different experimental studies have shown that EVs may have potential for therapeutic use as a liver-specific delivery method of different agents, taking advantage of their hepatocellular uptake through interactions with specific receptors. In this review, we focused on the most recent findings concerning the role of EVs as new structures mediating autocrine and paracrine intercellular communication in both ALD and NAFLD, as well as their potential use as biomarkers of disease severity and progression. Emerging therapeutic applications of EVs in these liver diseases were also examined, along with the potential for successful transition from bench to clinic.

## 1. Introduction

Knowledge of the pathobiology of extracellular vesicles (EVs) has expanded significantly in the last decade [1,2]. Indeed, significant advances have been made in delineating the mechanisms of assembly and release of EVs, as well as their subsequent membrane fusion with target cells [3,4]. Moreover, powerful analytical techniques have made possible the extensive characterization of the cargo of EVs, which includes myriad molecules including growth factors, metabolic enzymes, microRNAs and transcription factors, certain proteins, lipids, and metabolites, among others, that modulate intercellular and interorgan communication [3,5,6]. It is of note that high-throughput datasets of vesicular components are now available in public databases, which strongly supports EV research [7,8].

Novel insights into the biology of EVs show that these particles regulate critical biological functions and may act as contributors to disease pathogenesis, and may also serve as disease biomarkers, with the virtue of the relative simplicity of EV isolation from different biofluids [9]. In addition, EVs are gaining interest from a therapeutic point of view due to their potential as a unique drug delivery system [10].

In the field of hepatology, EVs have recently emerged as novel players in the pathogenesis and progression of several conditions [11,12,13], including the two most common liver diseases worldwide: nonalcoholic liver disease (NAFLD) and alcoholic liver disease (ALD) [14]. Specifically, recent studies point to a significant role of EVs in modulating injury, amplifying inflammation, and promoting liver fibrosis in both NAFLD and ALD [15,16,17]. Since information on this topic is dynamic and rapidly evolving, we aimed to provide an up-to-date overview of the current knowledge on the role of EVs in the context of both NAFLD and ALD, with emphasis on their potential diagnostic and therapeutic impact in these diseases. We excluded from this review data regarding EVs in liver cancer, since this has been recently reviewed elsewhere [18,19].

## 2. General Concepts of EVs in the Liver: EVs Biogenesis, Secretion, and Cargo

Details on the formation and secretion pathways of EVs have been recently reviewed elsewhere [15,20,21], and can also be found in other contributions to this special issue of Cells [22]. Only basic concepts are provided herein, as well as information on aspects that are of particular importance for liver physiology and pathophysiology.

In general, EVs are classified according to size and biogenetic pathway, such as exosomes, microvesicles, and apoptotic bodies [23]. Exosomes are bilayer lipid vesicles with a diameter of 30–150 nm that are derived from endosomal multivesicular bodies (MVBs) [15,23]. Their formation results from the invagination of the plasma membrane (early endosome) and the subsequent fusion of endocytic vesicles mediated by the endosomal sorting complex responsible for transport (ESCRTs) and other components (such as ceramides and tetraspanins) [24]. MVBs can release the intraluminal vesicles known as exosomes by the fusion of MVBs to the plasma membrane, a process mediated in part by Rab GTPases [25]. Microvesicles (MVs) have a diameter of 50–1000 nm and originate from the plasma membrane by budding and fission, followed by release into the extracellular space [15,23]. MVs contain a subset of cell surface proteins depending on the composition of the parental plasma membrane [26,27]. Apoptotic bodies have a diameter of 100–5000 nm, originate from the budding of cell membranes, and may contain nuclear material, which is quickly phagocytosed during programmed cell death [15,28]. Unlike exosomes and MVs, the role of apoptotic bodies is not related to intercellular communication. Therefore, when studying EVs under cellular damage conditions, apoptotic bodies are excluded [29].

The impact of EV research is highlighted by the existence of a dedicated scientific society (the International Society for Extracellular Vesicles, ISEV, https://www.isev.org/) that is constantly updating existent information on technical issues in order to homogenize the data being generated in different laboratories. To that end, the ISEV released a document entitled Minimal Information for Studies of Extracellular Vesicles (“MISEV”) Guidelines, to help researchers to better isolate and characterize their EV preparations, as well as suggesting protocols to document specific EV-associated functional activities [29]. At the present time, MISEV2018 does not propose molecular markers that could specifically characterize each EV subtype. However, understanding of existing subtypes and their associations with other cellular structures is an evolving matter and progress is expected in this regard [22]. Currently, the MISEV2018 guidelines suggest three categories of markers to be analyzed in all EV preparations: Categories 1 and 2, which demonstrate the presence of EVs, and Category 3, which assesses their purity from common contaminants. The latter recommends apolipoproteins A1/2 and B (APOA1/2, APOB), and albumin (ALB) as the best negative markers available at the present time.

All EVs transport a variety of bioactive molecules including cytoplasmic proteins, lipids, specific lipid-raft-interacting proteins, messenger RNA (mRNA), microRNA (miRNA), ribosomal RNA (rRNA), transfer RNA (tRNA), noncoding RNAs (ncRNAs), DNA, mitochondrial DNA (mtDNA), and metabolites [24,30]. Lipidomic analysis has shown that EVs, independently of their biogenesis, contain a multitude of lipids such as cholesterol, sphingomyelin, ceramide, saturated fatty acids, phosphatidylcholine, phosphatidylethanolamine, and phosphatidylserine [4,23,29,30]. In addition, proteomic analysis has shown that EVs contain different types of proteins, such as heat shock proteins (Hsp70 and Hsp90), tetraspanins (CD9, CD63, CD81, CD82), endosomal sorting complex proteins required for transport (Alix and Tsg101), receptors including epidermal growth factor receptor (EGFR), membrane trafficking proteins (GTPases, Flotillin and Annexins), cytoskeletal proteins (tubulin and actin), and cytosolic proteins [5,26]. It is worth noting that the cargo of EVs varies depending not only on their cellular origin, but also on the condition under which they are released (i.e., physiological vs. pathological).

**Figure 1:**
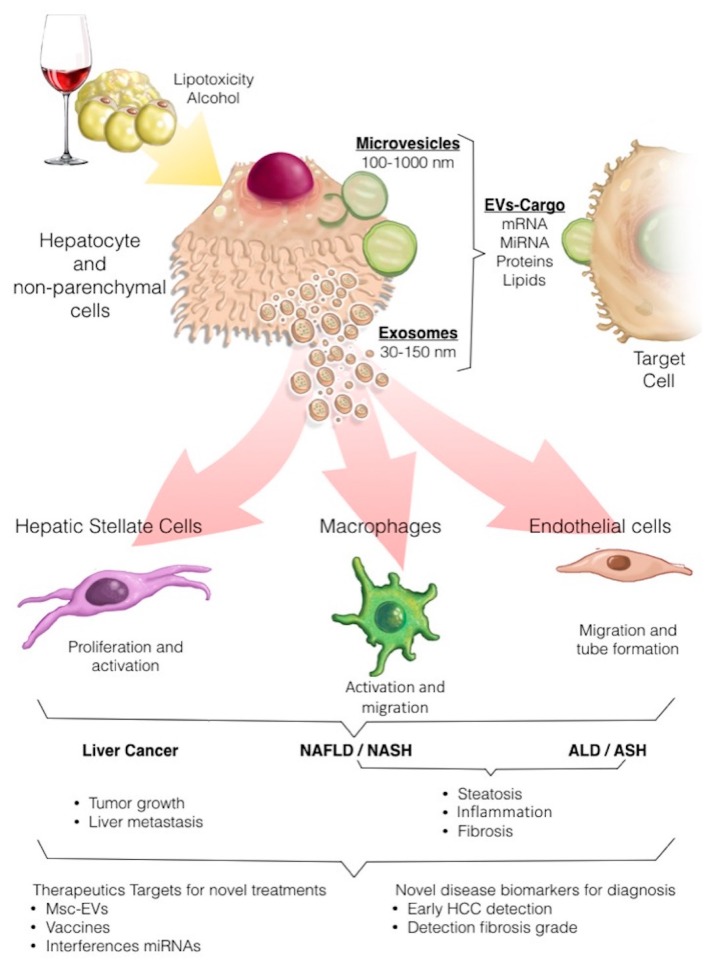
Extracellular vesicles (EVs) can be released by hepatocytes upon lipotoxic or alcohol-induced injury. EV cargoes include a multitude of molecules that can act on target cells, evoking inflammatory and fibrogenic events and promoting neoplastic transformation, thus contributing to the progression of both alcoholic liver disease (ALD) and nonalcoholic liver disease (NAFLD) to their inflammatory and more aggressive forms alcoholic steatohepatitis (ASH) and nonalcoholic steatohepatitis (NASH), respectively as well as to hepatocellular carcinoma (HCC development. EVs hold promise for both diagnosis (i.e., as biomarkers of disease severity or for diagnosis of HCC) and therapeutic purposes (i.e., therapeutic use of mesenchymal stem cell [MSC]-derived EVs, drug o miRNAs liver targeted therapies and anti-tumor vaccines).

In the liver, both parenchymal (hepatocytes) and non-parenchymal cells (e.g., hepatic stellate cells, endothelial, cholangiocytes, Kupfer cells, and liver endothelial cells) have been found to release EVs in both physiological and pathological states [20]. However, information on target cell repertoire, receptors, or other specific actions is still limited and incomplete. It has been shown that healthy hepatocytes produce limited numbers of exosomes containing proteins potentially relevant for cell survival, growth, and proliferation [31], whereas stressed hepatocytes boost exosome release [32] and enrich their content in specific proteins, lipids, and microRNAs that modulate the transcriptional program of neighboring hepatocytes and non-parenchymal cells, thus modulating inflammation and fibrosis, which are critical for the progression of liver diseases (Figure 1). Interestingly, recent evidence suggests that EVs from fat-laden hepatocytes can also signal to other organs such as adipose tissue, influencing adipogenesis and tissue remodeling [33].

## 3. EVs in Liver Pathobiology to Therapy

### 3.1. EVs and Liver Inflammation 

Hepatocellular damage determines the release of a number of signals into the extracellular environment that can contribute to tissue inflammation [34]. Some of these signals (collectively termed damage-associated molecular patterns (DAMPs)) are packaged in EVs and signal between hepatocytes and non-parenchymal cells such as liver-resident macrophages (Kupffer cells, KCs) [35]. Indeed, EVs may evoke the synthesis and release of proinflammatory cytokines such as pro-interleukin (IL)-1β and IL-6 [36] by KCs, thus contributing to local inflammation [37]. EVs released from hepatocytes can also promote the recruitment of additional immune cells (e.g., proinflammatory monocyte-derived macrophages) into the liver, maintaining and amplifying inflammation. EVs can also signal to endothelial cells and can contribute to vascular inflammation [38]. Furthermore, to add complexity, EVs can also be secreted from other cells and influence liver inflammation. In this regard, some evidence suggests that platelet-derived EVs may have proinflammatory effects in the liver, but this needs further confirmation [39,40]. Finally, it has also been shown that EVs are released from monocytic cells and induce polarization towards the anti-inflammatory M2 phenotype of neighboring naive monocytes by delivering cargo miR-27a, thus contributing to resolution of inflammation [41]. Other examples of modulation of immune response have been shown in studies of hepatitis B and C [42,43], which can participate in exacerbating liver injury when both viral infection and other injuring agents (i.e., alcohol) are at play.

Collectively, these findings suggest that EVs released from injured hepatocytes have an important role in modulating the inflammatory response during liver damage through intercellular communication between different cell types, with potential contributions from other cell-derived EVs [35]. Specific mechanisms involved in NAFLD and ALD are reviewed below.

### 3.2. EVs and Liver Fibrosis 

Persistent liver fibrogenesis and the development of cirrhosis are responsible for the liver-related morbidity and mortality associated with chronic liver diseases [44]. Activation or trans-differentiation of HSCs resulting in insoluble collagen deposition and distortion of the normal macro- and micro-anatomical structure of the liver is the major driver of liver fibrogenesis [45]. The role of paracrine signals originating from injured epithelial cells (hepatocytes) that can directly or indirectly induce HSC activation has been recognized in recent years [46]. In this regard, EVs seem to play a role, as shown by previous reported studies. Lipid-induced hepatocyte-derived EVs seem to regulate HSC activation by shuttling specific microRNAs (e.g., miR-128-3p) that suppress PPAR-γ expression in HSCs, leading to a marked increase of profibrogenic gene expression [47]. Other authors have shown that internalization of endothelial-derived exosomes by HSCs enhances cell migration in a process mediated by sphingosine 1-phosphate (S1P) [48]. Additionally, intercellular communication between quiescent and activated HSCs via exosomes can also modulate fibrosis. In this regard, a role for shuttled microRNA 214 (miR-214) in regulating the expression of alpha-smooth muscle actin and collagen in activated HSCs has been demonstrated [49,50]. Moreover, EVs derived from non-resident cells such as platelets or granulocytes have been shown to increase angiogenesis and to have procoagulant properties, thus promoting fibrogenesis [51,52]. These findings suggest that EVs appear to be key modulators in fibrosis, as signals from both parenchymal and non-parenchymal liver cells can either drive or slow down HSC activation. In addition, circulating EVs may be used as a biomarker of hepatic fibrosis and have potential implications for the development of novel anti-fibrotic targets [53], as reviewed in the following sections.

### 3.3. EVs as Biomarkers in Liver Diseases

Dynamic changes of EV generation in pathological conditions and the accessibility to measure and analyze them in biological samples (e.g., blood, urine, bile and other biofluids) make EVs good candidates to be used as disease biomarkers. The relative accessibility of testing and the ability to perform repeated measurements over time could facilitate early diagnosis, disease monitoring, and the development of personalized medicine. Furthermore, refinement of techniques allowing isolation and in-depth characterization of EV cargoes could lead to identification of disease-specific molecular signatures or profiles, providing ample opportunities for EVs to be used as suitable, non-invasive biomarkers [54].

EVs have been studied as potential biomarkers of liver injury in the settings of ALD, NAFLD, drug-induced liver disease, and cholangiopathies [13,27,55,56], as well as being considered diagnostic tools for liver cancer (e.g., hepatocellular carcinoma and cholangiocarcinoma) [19,56]. In this regard, determination of the number of circulating EVs and nucleic-acid based, lipid-based, and protein-based diagnostics have been applied to measure EVs enriched in liver-derived DNA, microRNAs, lipids, or proteins [13,27]. However, before implementing EV-based biomarkers in clinical settings, standardization of sample processing (e.g., collection, transportation, storage, and handling) and assay systems is needed, as well as large replicative studies to allow EV molecular signatures to be conclusively linked to specific liver diseases. Recent advances related to NAFLD and ALD are reviewed below.

### 3.4. Therapeutic Potential of EVs 

From a therapeutic perspective, EVs, either unmodified or engineered, can be utilized for therapeutic purposes [57]. With regard to liver diseases, efforts have been focused on two major areas: (a) the use of EVs as delivery vehicles of drugs to the liver [58] and (b) the use of EVs themselves as therapeutic agents to stimulate liver regeneration, modulate inflammation, reduce liver fibrosis, or halt hepatocarcinogenesis [59,60]. The former approach involves the use of different techniques to load EVs with a desired cargo (e.g., miRNA, siRNA, chemotherapeutic agents) to act as a “Trojan horse” to target cells. Theoretically, the membrane properties of EVs allow organ- and cell-specific delivery, immune-evasion, and targeting of distinct intracellular trafficking pathways. Moreover, EVs could be modified to improve liver-specific targeting through complex chemical methods (e.g., click chemistry) to alter their surface to favor targeting, or loading them with desired therapeutic agents using a variety of techniques (e.g., electroporation, saponin-induced pore formation, hypotonic dialysis, and others) [60]. Larger EVs (e.g., microvesicles) could be more suitable for therapeutic purposes, as they may allow better drug loading and biodistribution [61]. In spite of these advantages, a number of challenges related to the manufacturing of EVs (i.e., production, coating, loading, etc.) still need to be solved before controlled clinical studies can be carried out [62,63].

Regarding the use of EVs as therapeutic agents, most of the available evidence has been generated using mesenchymal stem cell (MSC)-derived EVs obtained from human umbilical cords or human embryos that have been tested in numerous preclinical liver disease models (e.g., carbon tetrachloride, thioacetamide, D-galactosamine/TNF-α-induced lethal hepatic failure, and bile duct ligation) with promising results [11,53]. Data for NAFLD and ALD are more limited and are reviewed in the corresponding sections below.

A third, and still nascent, approach related to EV-based therapy is based on the concept that interfering with EV secretion or uptake may attenuate harmful effects on target cells [11,64]. In this regard, several pharmacological agents are being explored that have been shown to inhibit EV trafficking, modify lipid metabolism, or decrease EV secretion. However, the complexity of EV biogenesis poses significant challenges to the development of specific agents able to selectively block EV production (see Reference [64] for an in-depth review of this topic).

## 4. EVs in NAFLD and ALD

### 4.1. EVs in NAFLD 

A number of studies have demonstrated a role of EVs in both the pathogenesis and progression of NAFLD [14,15]. Triggering of inflammation and fibrosis development are key for progression from isolated steatosis (also referred as NAFL or non-NASH fatty liver) to nonalcoholic steatohepatitis (NASH), which is hallmarked by the presence of hepatocyte ballooning as a reflection of ongoing liver injury and death [36]. Data from different diet-induced animal models of NASH have shown that EV concentration increases with disease progression in a time-dependent manner [17,65,66]. This seems to be a response to the accumulation of toxic lipids and their downstream mediators in the liver, which increase the capacity of hepatocytes to form and release different types of EVs [37,65,66,67]. In vitro treatment of hepatocytes with non-esterified fatty acids evokes the release of EVs containing numerous molecules including C-X-C motif ligand 10 (CXCL10), sphingosine-1-phosphate (S1P), mitochondrial DNA (mtDNA), micro-RNAs, ceramides, and tumor necrosis factor-related apoptosis-inducing ligand (TRAIL) (15). These molecules may amplify inflammation through multiple mechanisms such as macrophage activation and monocyte chemotaxis, as well as inflammasome activation and modulation of the NF-κB pathway in target cells [67,68]. As mentioned earlier, EVs may be released by different mechanisms including a caspase-3-dependent mechanism [66] or activation of death receptor 5 (DR5) in hepatocytes. Hepatocyte-derived EVs are able to induce expression of proinflammatory cytokines and promote M1 polarization of hepatic macrophages [37,69]. CXCL10-bearing EVs can also serve as chemotactic stimuli for macrophages, as shown recently [67]. Moreover, EVs released from hepatocytes can contribute to hepatic recruitment of monocyte-derived macrophages by promoting monocyte adhesion via integrin β1 (ITGβ1)-dependent mechanisms, as shown in murine NASH [70]. Additional stimuli can also stimulate EV release from hepatocytes. In this regard, it has been observed that hypoxia determines that fat-laden hepatocytes release EVs able to signal KCs, evoking proinflammatory phenotypes in these cells, a phenomenon that may explain the aggravating effect of obstructive sleep apnea syndrome on NAFLD [71]. Thus, it seems clear that lipotoxic injury of hepatocytes determines EV release, promoting inflammation through activation and recruitment of macrophages [14], with clear implications for the triggering of inflammation in NAFLD/NASH [36]. In addition, hepatocyte-derived EVs may promote HSC activation [47,72] in experimental models of NAFLD/NASH. Interestingly, both mouse and human HSCs release EVs that target hepatocytes and HSCs themselves [49,50,73]. Collectively, these data implicate EVs as one aspect of the cellular events triggering hepatic fibrogenesis, a key process in NAFLD progression [74]. In addition to signaling to macrophages and HSCs, EVs may act on endothelial cells [38,66], promoting vascular inflammation with potential implications for NAFLD-related atherosclerosis. In this regard, it has been shown that EVs carrying miR-1 as cargo mediate proinflammatory effects in endothelial cells in mice via downregulation of KLF4 and activation of the NF-κB pathway [38]. Finally, other organ-derived EVs (e.g., visceral-adipose-tissue-derived exosomes) can contribute to NAFLD pathogenesis and progression and influence fibrogenic pathways in both hepatocytes and HSCs [75], underscoring the role of other insulin-sensitive organs in NAFLD.

Studies carried out in mouse models of NASH have shown that total circulating EVs and particularly hepatocyte-derived EVs are elevated early in the disease process, while other cell-derived EVs (e.g., macrophage- and neutrophil-derived EVs) appear in the circulation later, likely reflecting the ongoing inflammatory process [65,76]. Proteomic profiling of circulating EVs in experimental NAFLD has been demonstrated to allow differentiation between NAFLD vs. control animals [17]. These findings underscore the potential of EVs as minimally invasive biomarkers for NAFLD [77], which are urgently needed for clinical trials and in clinical settings. Since circulating EVs (mainly exosomes) are also increased in human NAFLD and have been found by some authors to correlate with disease histological features [78], EV analysis in serum involving quantitative and qualitative determinations (including cell surface marker assessment and measurement of different cargoes (e.g., proteins, lipids, and microRNAs)) is now a focus of intense research. Several studies have been published in this regard, showing that the number of CD14+ and CD16+ EVs is inversely associated with the severity of NAFLD-related liver fibrosis, while also increasing the diagnostic capability of the enhanced liver fibrosis score (LFS) in patients with NAFLD (AUC: 0.948 and 0.967 for CD14+ and CD16+ EVs, respectively, vs. 0.915 for LFS alone) [79]). Other efforts include detection of circulating EVs containing C16:0 ceramide- and S1P-enriched lipid species, which progressively increase in the plasma of obese patients with simple steatosis and in NASH patients with early fibrosis [65]. Unfortunately, the diagnostic accuracy of these determinations remains incompletely explored in the field of NAFLD/NASH, and rigorous validation of this approach is needed [13,77]. Moreover, significant challenges remain regarding the isolation, reproducibility, and definition of normal controls [13,80].

Therapeutic efforts involving EVs in the field of NAFLD/NASH are nascent. Attempts to halt inflammation and fibrosis in rodent models of NAFLD/NASH using EVs as therapeutic agents have been published recently. EVs obtained from amnion-derived MSCs (AMSCs) were used to treat rats with either NASH or liver fibrosis induced by the hepatic toxicant CCL4. AMSC-EVs were given intravenously in one or two doses, and amelioration of inflammation and fibrogenesis was observed [81]. More recently, human liver stem cell (HLSC)-derived EVs have been used to treat mice with diet-induced steatohepatitis [82]. The authors found that EV-HLSC treatment significantly downregulated hepatic profibrotic and proinflammatory gene expression and ameliorated the histological abnormalities in mice with NASH. Proteomic analysis of EV-HLSCs showed that their cargo included various anti-inflammatory proteins such as Interleukin-10, which may have contributed to the observed beneficial effects. In addition, although yet not explored in the field of NAFLD, the use of MSC-derived EVs to arrest fibrogenesis [53,83] holds promise to treat this condition, as fibrosis is one of the most important determinants of survival. These results underscore the concept that EVs can be exploited for therapy in NAFLD/NASH. 

### 4.2. EVs in ALD 

Recent studies have focused on the role of EVs in ALD [11,14,84,85]. Both hepatocyte- and monocyte-derived EVs have been postulated to regulate macrophage differentiation, thereby promoting inflammation in alcoholic hepatitis (AH) [24,41,55,72,86]. Several molecules have been proposed to be responsible for EV-mediated cell-to-cell signaling, including miRNAs (in particular, miR-122 and miR-155) [41,85,87,88,89,90,91,92]. Also, the CD40 ligand was proposed as an EV cargo that could promote macrophage activation in vitro and in vivo in experimental models of AH [85]. Another study involving a mouse model of ALD using gastric infusion of ethanol, found that circulating EVs released by hepatocytes contain a microRNA barcode (let7f, miR-29a, and miR-340), which was specific for alcohol-related liver injury [16].

From a clinical point of view, there is currently no biomarker able to assess the early stages of ALD. EVs have been shown to correlate with the diagnosis and prognosis of alcoholic hepatitis [93]. Additionally, EVs have been proposed as potential biomarkers to differentiate mild and severe forms of ALD, and their cargo, such as sphingolipids, could be used to discriminate between different liver disease etiologies [93]. Early identification of these subjects might lead to timely intervention in the disease process. At present, the diagnosis of AH relies on a history of alcohol consumption in a compatible clinical scenario. A study conducted in trauma patients showed that two microRNAs (miR-122 and let7f) were increased only in the circulating EVs obtained from alcohol drinkers with evidence of liver injury [94]. This suggest that these microRNAs could serve to detect “at-risk” populations for ALD. With regard to AH, the most severe form of the disease, at present time there are no biomarkers which permit the early diagnosis of AH, or screening tests that can anticipate those at risk for more severe manifestations of AH. Identification of this group of patients is particularly important since there is no effective therapy for severe AH, and efforts to avoid it could be implemented. Several clinical prognostic markers have been proposed, but the variables used reflect the severity of liver disease. These indices (e.g., the Maddrey discriminant function, model for end-stage liver disease (MELD), and the Lille score) are based on the non-specific biochemical assessment of liver and renal function and rely heavily on serum total bilirubin, prothrombin time, and creatinine [95]. The reliance on bilirubin limits their diagnostic utility, as specificity is confounded by hyperbilirubinemia in co-existent cholestatic liver diseases such as drug-induced liver disease, or cholestatic hepatitis such as primary sclerosing cholangitis and primary biliary cholangitis. None of these prognostic scoring methods utilize a pathophysiologically validated biomarker that reflects the underlying molecular and signaling mechanisms of the disease. Furthermore, the inflammatory response is predictive of mortality but is not taken into account in mathematical models. They can identify subjects at highest mortality risk with an area under the receiver operating curve (AUROC) of under 0.8; ideal survival models should have an AUROC > 0.8 [96]. The reason these scores are imperfect may be that they are not based on the pathophysiological mechanisms that mediate liver injury. Liver biopsy, the gold standard for diagnosis of AH, remains underutilized due to the concurrent coagulopathy, which greatly increases the risk of biopsy-related complications [97]. Liver-biopsy-based hepatic histology score has an AUROC of 0.73 in predicting 90 day mortality [96]. In this regard, the use of EVs as biomarker in AH is promising. Thus, it was recently reported that the total number of EVs was significantly increased in patients with AH and that two microRNAs (e.g., miRNA-192 and miRNA-30a) were significantly increased in the plasma of subjects with AH [86]. Additionally, a very recent study showed that circulating EVs carrying a sphingolipid cargo might be helpful for the diagnosis of AH, and may allow dynamic risk profiling of these patients. [98]. Most recently, EVs have been used as a surrogate marker of improvement in clinical trials of patients with AH [99]. Further studies will be required to validate EVs as a biomarker for AH diagnosis and/or prognosis.

## 5. Concluding Remarks and Future Perspectives 

The field of EVs in fatty liver diseases is rapidly evolving as the important functions of these particles in cell-to-cell communication and in the pathogenesis of both ALD and NAFLD, the most prevalent liver diseases worldwide, are unveiled. The release of large quantities of EVs by stressed/damaged hepatocytes contributes to inflammation, fibrogenesis, and angiogenesis, fueling liver disease progression and provides opportunities for intervention. The identification of specific molecular signatures of released EVs is promising in the search for disease-specific biomarkers, although more data are needed to validate these markers in larger cohorts and in a rigorous manner. EVs may have potential for therapeutic use, but this field is still nascent. More research is needed for a successful transition of current EV knowledge from the bench to the clinic.

## References

[1] Stahl P.D., Raposo G. (2019). Extracellular Vesicles: Exosomes and Microvesicles, Integrators of Homeostasis. Physiology.

[2] Cocucci E., Racchetti G., Meldolesi J. (2009). Shedding microvesicles: Artefacts no more. Trends Cell Biol..

[3] Abels E.R., Breakefield X.O. (2016). Introduction to Extracellular Vesicles: Biogenesis, RNA Cargo Selection, Content, Release, and Uptake. Cell. Mol. Neurobiol..

[4] van Niel G., D’Angelo G., Raposo G. (2018). Shedding light on the cell biology of extracellular vesicles. Nat. Rev. Mol. Cell Biol..

[5] Kowal J., Arras G., Colombo M., Jouve M., Morath J.P., Primdal-Bengtson B., Dingli F., Loew D., Tkach M., Théry C. (2016). Proteomic comparison defines novel markers to characterize heterogeneous populations of extracellular vesicle subtypes. Proc. Natl. Acad. Sci. USA.

[6] Zivko C., Fuhrmann G., Luciani P. (2020). Liver-derived extracellular vesicles: A cell by cell overview to isolation and characterization practices. Biochim. Biophys. Acta Gen. Subj..

[7] Kim D.K., Lee J., Kim S.R., Choi D.S., Yoon Y.J., Kim J.H., Go G., Nhung D., Hong K., Jang S.C. (2015). EVpedia: A community web portal for extracellular vesicles research. Bioinformatics.

[8] Kim D.K., Kang B., Kim O.Y., Choi D.S., Lee J., Kim S.R., Go G., Yoon Y.J., Kim J.H., Jang S.C. (2013). EVpedia: An integrated database of high-throughput data for systemic analyses of extracellular vesicles. J. Extracell. Vesicles.

[9] Shao H., Im H., Castro C.M., Breakefield X., Weissleder R., Lee H. (2018). New Technologies for Analysis of Extracellular Vesicles. Chem. Rev..

[10] Maas S.L.N., Breakefield X.O., Weaver A.M. (2017). Extracellular Vesicles: Unique Intercellular Delivery Vehicles. Trends Cell Biol..

[11] Urban S.K., Mocan T., Sanger H., Lukacs-Kornek V., Kornek M. (2019). Extracellular Vesicles in Liver Diseases: Diagnostic, Prognostic, and Therapeutic Application. Semin. Liver Dis..

[12] Lemoinne S., Thabut D., Housset C., Moreau R., Valla D., Boulanger C.M., Rautou P.E. (2014). The emerging roles of microvesicles in liver diseases. Nat. Rev. Gastroenterol. Hepatol..

[13] Szabo G., Momen-Heravi F. (2017). Extracellular vesicles in liver disease and potential as biomarkers and therapeutic targets. Nat. Rev. Gastroenterol. Hepatol..

[14] Eguchi A., Feldstein A.E. (2018). Extracellular vesicles in non-alcoholic and alcoholic fatty liver diseases. Liver Res..

[15] Malhi H. (2019). Emerging role of extracellular vesicles in liver diseases. Am. J. Physiol. Gastrointest. Liver Physiol..

[16] Eguchi A., Lazaro R.G., Wang J., Kim J., Povero D., Willliams B., Ho S.B., Stärkel P., Schnabl B., Ohno-Machado L. (2017). Extracellular vesicles released by hepatocytes from gastric infusion model of alcoholic liver disease contain a MicroRNA barcode that can be detected in blood. Hepatology.

[17] Povero D., Eguchi A., Li H., Johnson C.D., Papouchado B.G., Wree A., Messer K., Feldstein A.E. (2014). Circulating extracellular vesicles with specific proteome and liver microRNAs are potential biomarkers for liver injury in experimental fatty liver disease. PLoS ONE.

[18] Xie F., Feng S., Yang H., Mao Y. (2019). Extracellular vesicles in hepatocellular cancer and cholangiocarcinoma. Ann. Transl. Med..

[19] Sasaki R., Kanda T., Yokosuka O., Kato N., Matsuoka S., Moriyama M. (2019). Exosomes and Hepatocellular Carcinoma: From Bench to Bedside. Int. J. Mol. Sci..

[20] Sung S., Kim J., Jung Y. (2018). Liver-Derived Exosomes and Their Implications in Liver Pathobiology. Int. J. Mol. Sci..

[21] Latifkar A., Hur Y.H., Sanchez J.C., Cerione R.A., Antonyak M.A. (2019). New insights into extracellular vesicle biogenesis and function. J. Cell Sci..

[22] Doyle L.M., Wang M.Z. (2019). Overview of Extracellular Vesicles, Their Origin, Composition, Purpose, and Methods for Exosome Isolation and Analysis. Cells.

[23] Moran L., Cubero F.J. (2018). Extracellular vesicles in liver disease and beyond. World J. Gastroenterol..

[24] Devhare P.B., Ray R.B. (2018). Extracellular vesicles: Novel mediator for cell to cell communications in liver pathogenesis. Mol. Aspects Med..

[25] Hirsova P., Ibrahim S.H., Verma V.K., Morton L.A., Shah V.H., LaRusso N.F., Gores G.J., Malhi H. (2016). Extracellular vesicles in liver pathobiology: Small particles with big impact. Hepatology.

[26] Haraszti R.A., Didiot M.C., Sapp E., Leszyk J., Shaffer S.A., Rockwell H.E., Gao F., Narain N.R., DiFiglia M., Kiebish M.A. (2016). High-resolution proteomic and lipidomic analysis of exosomes and microvesicles from different cell sources. J. Extracell. Vesicles.

[27] Banales J.M., Feldstein A.E., Sanger H., Lukacs-Kornek V., Szabo G., Kornek M. (2019). Extracellular Vesicles in Liver Diseases: Meeting Report from the International Liver Congress 2018. Hepatol. Commun..

[28] Aizawa S., Brar G., Tsukamoto H. (2019). Cell Death and Liver Disease. Gut Liver.

[29] Théry C., Witwer K.W., Aikawa E., Alcaraz M.J., Anderson J.D., Andriantsitohaina R., Antoniou A., Arab T., Archer F., Atkin-Smith G.K. (2018). Minimal information for studies of extracellular vesicles 2018 (MISEV2018): A position statement of the International Society for Extracellular Vesicles and update of the MISEV2014 guidelines. J. Extracell. Vesicles.

[30] Royo F., Gil-Carton D., Gonzalez E., Mleczko J., Palomo L., Perez-Cormenzana M., Mayo R., Alonso C., Falcon-Perez J.M. (2019). Differences in the metabolite composition and mechanical properties of extracellular vesicles secreted by hepatic cellular models. J. Extracell. Vesicles.

[31] Nojima H., Freeman C.M., Schuster R.M., Japtok L., Kleuser B., Edwards M.J., Gulbins E., Lentsch A.B. (2016). Hepatocyte exosomes mediate liver repair and regeneration via sphingosine-1-phosphate. J. Hepatol..

[32] Chen L., Chen R., Kemper S., Brigstock D.R. (2018). Pathways of production and delivery of hepatocyte exosomes. J. Cell Commun. Signal..

[33] Zhao Y., Zhao M.F., Jiang S., Wu J., Liu J., Yuan X.W., Shen D., Zhang J.Z., Zhou N., He J. (2020). Liver governs adipose remodelling via extracellular vesicles in response to lipid overload. Nat. Commun..

[34] Arrese M., Cabrera D., Kalergis A.M., Feldstein A.E. (2016). Innate Immunity and Inflammation in NAFLD/NASH. Dig. Dis. Sci..

[35] Sato K., Kennedy L., Liangpunsakul S., Kusumanchi P., Yang Z., Meng F., Glaser S., Francis H., Alpini G. (2019). Intercellular Communication between Hepatic Cells in Liver Diseases. Int. J. Mol. Sci..

[36] Schuster S., Cabrera D., Arrese M., Feldstein A.E. (2018). Triggering and resolution of inflammation in NASH. Nat. Rev. Gastroenterol. Hepatol..

[37] Hirsova P., Ibrahim S.H., Krishnan A., Verma V.K., Bronk S.F., Werneburg N.W., Charlton M.R., Shah V.H., Malhi H., Gores G.J. (2016). Lipid-Induced Signaling Causes Release of Inflammatory Extracellular Vesicles From Hepatocytes. Gastroenterology.

[38] Jiang F., Chen Q., Wang W., Ling Y., Yan Y., Xia P. (2020). Hepatocyte-derived extracellular vesicles promote endothelial inflammation and atherogenesis via microRNA-1. J. Hepatol..

[39] Antwi-Baffour S., Adjei J., Aryeh C., Kyeremeh R., Kyei F., Seidu M.A. (2015). Understanding the biosynthesis of platelets-derived extracellular vesicles. Immun. Inflamm. Dis..

[40] Balaphas A., Meyer J., Sadoul K., Fontana P., Morel P., Gonelle-Gispert C., Bühler L.H. (2019). Platelets and Platelet-Derived Extracellular Vesicles in Liver Physiology and Disease. Hepatol. Commun..

[41] Saha B., Momen-Heravi F., Kodys K., Szabo G. (2016). MicroRNA Cargo of Extracellular Vesicles from Alcohol-exposed Monocytes Signals Naive Monocytes to Differentiate into M2 Macrophages. J. Biol. Chem..

[42] Grünvogel O., Colasanti O., Lee J.Y., Klöss V., Belouzard S., Reustle A., Esser-Nobis K., Hesebeck-Brinckmann J., Mutz P., Hoffmann K. (2018). Secretion of Hepatitis C Virus Replication Intermediates Reduces Activation of Toll-Like Receptor 3 in Hepatocytes. Gastroenterology.

[43] Li S., Li S., Wu S., Chen L. (2019). Exosomes Modulate the Viral Replication and Host Immune Responses in HBV Infection. Biomed. Res. Int..

[44] Lee Y.A., Wallace M.C., Friedman S.L. (2015). Pathobiology of liver fibrosis: A translational success story. Gut.

[45] Friedman S.L. (2008). Hepatic stellate cells: Protean, multifunctional, and enigmatic cells of the liver. Physiol. Rev..

[46] Higashi T., Friedman S.L., Hoshida Y. (2017). Hepatic stellate cells as key target in liver fibrosis. Adv Drug Deliv Rev..

[47] Povero D., Panera N., Eguchi A., Johnson C.D., Papouchado B.G., de Araujo Horcel L., Pinatel E.M., Alisi A., Nobili V., Feldstein A.E. (2015). Lipid-induced hepatocyte-derived extracellular vesicles regulate hepatic stellate cell via microRNAs targeting PPAR-gamma. Cell. Mol. Gastroenterol. Hepatol..

[48] Wang R., Ding Q., Yaqoob U., de Assuncao T.M., Verma V.K., Hirsova P., Cao S., Mukhopadhyay D., Huebert R.C., Shah V.H. (2015). Exosome Adherence and Internalization by Hepatic Stellate Cells Triggers Sphingosine 1-Phosphate-dependent Migration. J. Biol. Chem..

[49] Chen L., Charrier A., Zhou Y., Chen R., Yu B., Agarwal K., Tsukamoto H., Lee L.J., Paulaitis M.E., Brigstock D.R. (2014). Epigenetic regulation of connective tissue growth factor by MicroRNA-214 delivery in exosomes from mouse or human hepatic stellate cells. Hepatology.

[50] Charrier A., Chen R., Chen L., Kemper S., Hattori T., Takigawa M., Brigstock D.R. (2014). Exosomes mediate intercellular transfer of pro-fibrogenic connective tissue growth factor (CCN2) between hepatic stellate cells, the principal fibrotic cells in the liver. Surgery.

[51] Owens A.P., Mackman N. (2011). Microparticles in hemostasis and thrombosis. Circ. Res..

[52] Valla D.C. (2008). Thrombosis and anticoagulation in liver disease. Hepatology.

[53] Chen L., Brenner D.A., Kisseleva T. (2019). Combatting Fibrosis: Exosome-Based Therapies in the Regression of Liver Fibrosis. Hepatol. Commun..

[54] Clayton A., Buschmann D., Byrd J.B., Carter D.R., Cheng L., Compton C., Daaboul G., Devitt A., Falcon-Perez J.M., Gardiner C. (2018). Summary of the ISEV workshop on extracellular vesicles as disease biomarkers, held in Birmingham, UK, during December 2017. J. Extracell. Vesicles.

[55] Cho Y.E., Song B.J., Akbar M., Baek M.C. (2018). Extracellular vesicles as potential biomarkers for alcohol- and drug-induced liver injury and their therapeutic applications. Pharmacol. Ther..

[56] Arbelaiz A., Azkargorta M., Krawczyk M., Santos-Laso A., Lapitz A., Perugorria M.J., Erice O., Gonzalez E., Jimenez-Agüero R., Lacasta A. (2017). Serum extracellular vesicles contain protein biomarkers for primary sclerosing cholangitis and cholangiocarcinoma. Hepatology.

[57] Murphy D.E., de Jong O.G., Brouwer M., Wood M.J., Lavieu G., Schiffelers R.M., Vader P. (2019). Extracellular vesicle-based therapeutics: Natural versus engineered targeting and trafficking. Exp. Mol. Med..

[58] Villa F., Quarto R., Tasso R. (2019). Extracellular Vesicles as Natural, Safe and Efficient Drug Delivery Systems. Pharmaceutics.

[59] Balaphas A., Meyer J., Sadoul R., Morel P., Gonelle-Gispert C., Buhler L.H. (2019). Extracellular vesicles: Future diagnostic and therapeutic tools for liver disease and regeneration. Liver Int..

[60] Borrelli D.A., Yankson K., Shukla N., Vilanilam G., Ticer T., Wolfram J. (2018). Extracellular vesicle therapeutics for liver disease. J. Control. Release.

[61] Kanada M., Bachmann M.H., Hardy J.W., Frimannson D.O., Bronsart L., Wang A., Sylvester M.D., Schmidt T.L., Kaspar R.L., Butte M.J. (2015). Differential fates of biomolecules delivered to target cells via extracellular vesicles. Proc. Natl. Acad. Sci. USA.

[62] Gao J., Dong X., Wang Z. (2019). Generation, purification and engineering of extracellular vesicles and their biomedical applications. Methods.

[63] Patel D.B., Santoro M., Born L.J., Fisher J.P., Jay S.M. (2018). Towards rationally designed biomanufacturing of therapeutic extracellular vesicles: Impact of the bioproduction microenvironment. Biotechnol. Adv..

[64] Catalano M., O’Driscoll L. (2019). Inhibiting extracellular vesicles formation and release: A review of EV inhibitors. J. Extracell. Vesicles.

[65] Kakazu E., Mauer A.S., Yin M., Malhi H. (2016). Hepatocytes release ceramide-enriched pro-inflammatory extracellular vesicles in an IRE1alpha-dependent manner. J. Lipid Res..

[66] Povero D., Eguchi A., Niesman I.R., Andronikou N., du Jeu X.D., Mulya A., Berk M., Lazic M., Thapaliya S., Parola M. (2013). Lipid-induced toxicity stimulates hepatocytes to release angiogenic microparticles that require Vanin-1 for uptake by endothelial cells. Sci. Signal..

[67] Ibrahim S.H., Hirsova P., Tomita K., Bronk S.F., Werneburg N.W., Harrison S.A., Goodfellow V.S., Malhi H., Gores G.J. (2016). Mixed lineage kinase 3 mediates release of C-X-C motif ligand 10-bearing chemotactic extracellular vesicles from lipotoxic hepatocytes. Hepatology.

[68] Cannito S., Morello E., Bocca C., Foglia B., Benetti E., Novo E., Chiazza F., Rogazzo M., Fantozzi R., Povero D. (2017). Microvesicles released from fat-laden cells promote activation of hepatocellular NLRP3 inflammasome: A pro-inflammatory link between lipotoxicity and non-alcoholic steatohepatitis. PLoS ONE.

[69] Liu X.L., Pan Q., Cao H.X., Xin F.Z., Zhao Z.H., Yang R.X., Zeng J., Zhou H., Fan J.G. (2019). Lipotoxic Hepatocyte-Derived Exosomal miR-192-5p Activates Macrophages via Rictor/Akt/FoxO1 Signaling in NAFLD. Hepatology.

[70] Guo Q., Furuta K., Lucien F., Gutierrez Sanchez L.H., Hirsova P., Krishnan A., Kabashima A., Pavelko K.D., Madden B., Alhuwaish H. (2019). Integrin beta1-enriched extracellular vesicles mediate monocyte adhesion and promote liver inflammation in murine NASH. J. Hepatol..

[71] Hernandez A., Geng Y., Sepulveda R., Solis N., Torres J., Arab J.P., Barrera F., Cabrera D., Moshage H., Arrese M. (2020). Chemical Hypoxia induces pro-inflammatory signals in fat-laden hepatocytes and contributes to cellular crosstalk with Kupffer cells through extracellular vesicles. BBA Mol. Basis Dis..

[72] Lee Y.S., Kim S.Y., Ko E., Lee J.H., Yi H.S., Yoo Y.J., Je J., Suh S.J., Jung Y.K., Kim J.H. (2017). Exosomes derived from palmitic acid-treated hepatocytes induce fibrotic activation of hepatic stellate cells. Sci. Rep..

[73] Chen L., Chen R., Kemper S., Charrier A., Brigstock D.R. (2015). Suppression of fibrogenic signaling in hepatic stellate cells by Twist1-dependent microRNA-214 expression: Role of exosomes in horizontal transfer of Twist1. Am. J. Physiol. Gastrointest. Liver Physiol..

[74] Schuppan D., Surabattula R., Wang X.Y. (2018). Determinants of fibrosis progression and regression in NASH. J. Hepatol..

[75] Koeck E.S., Iordanskaia T., Sevilla S., Ferrante S.C., Hubal M.J., Freishtat R.J., Nadler E.P. (2014). Adipocyte exosomes induce transforming growth factor beta pathway dysregulation in hepatocytes: A novel paradigm for obesity-related liver disease. J. Surg. Res..

[76] Li J., Liu H., Mauer A.S., Lucien F., Raiter A., Bandla H., Mounajjed T., Yin Z., Glaser K.J., Yin M. (2019). Characterization of Cellular Sources and Circulating Levels of Extracellular Vesicles in a Dietary Murine Model of Nonalcoholic Steatohepatitis. Hepatol. Commun..

[77] Ban L.A., Shackel N.A., McLennan S.V. (2016). Extracellular Vesicles: A New Frontier in Biomarker Discovery for Non-Alcoholic Fatty Liver Disease. Int. J. Mol. Sci..

[78] Kornek M., Lynch M., Mehta S.H., Lai M., Exley M., Afdhal N.H., Schuppan D. (2012). Circulating microparticles as disease-specific biomarkers of severity of inflammation in patients with hepatitis C or nonalcoholic steatohepatitis. Gastroenterology.

[79] Welsh J.A., Scorletti E., Clough G.F., Englyst N.A., Byrne C.D. (2018). Leukocyte extracellular vesicle concentration is inversely associated with liver fibrosis severity in NAFLD. J. Leukoc. Biol..

[80] Momen-Heravi F., Szabo G., Irwin M., Arias M.D., Harvey J., Alter M.D., James L., Boyer M.D., David E., Cohen M.D., David A., Shafritz M.D. (2020). Extracellular Vesicles and Exosomes: Biology and Pathobiology. The Liver: Biology and Pathobiology.

[81] Ohara M., Ohnishi S., Hosono H., Yamamoto K., Yuyama K., Nakamura H., Fu Q., Maehara O., Suda G., Sakamoto N. (2018). Extracellular Vesicles from Amnion-Derived Mesenchymal Stem Cells Ameliorate Hepatic Inflammation and Fibrosis in Rats. Stem Cells Int..

[82] Bruno S., Pasquino C., Sanchez M.B., Tapparo M., Figliolini F., Grange C., Chiabotto G., Cedrino M., Deregibus M.C., Tetta C. (2019). HLSC-Derived Extracellular Vesicles Attenuate Liver Fibrosis and Inflammation in a Murine Model of Non-alcoholic Steatohepatitis. Mol. Ther..

[83] Chen L., Chen R., Kemper S., Cong M., You H., Brigstock D.R. (2018). Therapeutic effects of serum extracellular vesicles in liver fibrosis. J. Extracell. Vesicles.

[84] Rahman M.A., Patters B.J., Kodidela S., Kumar S. (2019). Extracellular Vesicles: Intercellular Mediators in Alcohol-Induced Pathologies. J. Neuroimmune Pharmacol..

[85] Verma V.K., Li H., Wang R., Hirsova P., Mushref M., Liu Y., Cao S., Contreras P.C., Malhi H., Kamath P.S. (2016). Alcohol stimulates macrophage activation through caspase-dependent hepatocyte derived release of CD40L containing extracellular vesicles. J. Hepatol..

[86] Momen-Heravi F., Saha B., Kodys K., Catalano D., Satishchandran A., Szabo G. (2015). Increased number of circulating exosomes and their microRNA cargos are potential novel biomarkers in alcoholic hepatitis. J. Transl. Med..

[87] Bala S., Petrasek J., Mundkur S., Catalano D., Levin I., Ward J., Alao H., Kodys K., Szabo G. (2012). Circulating microRNAs in exosomes indicate hepatocyte injury and inflammation in alcoholic, drug-induced, and inflammatory liver diseases. Hepatology.

[88] Momen-Heravi F., Bala S., Kodys K., Szabo G. (2015). Exosomes derived from alcohol-treated hepatocytes horizontally transfer liver specific miRNA-122 and sensitize monocytes to LPS. Sci. Rep..

[89] Csak T., Bala S., Lippai D., Satishchandran A., Catalano D., Kodys K., Szabo G. (2015). microRNA-122 regulates hypoxia-inducible factor-1 and vimentin in hepatocytes and correlates with fibrosis in diet-induced steatohepatitis. Liver Int..

[90] Li H.D., Du X.S., Huang H.M., Chen X., Yang Y., Huang C., Meng X.M., Li J. (2019). Noncoding RNAs in alcoholic liver disease. J. Cell. Physiol..

[91] Lamichhane T.N., Leung C.A., Douti L.Y., Jay S.M. (2017). Ethanol Induces Enhanced Vascularization Bioactivity of Endothelial Cell-Derived Extracellular Vesicles via Regulation of MicroRNAs and Long Non-Coding RNAs. Sci. Rep..

[92] Brandon-Warner E., Feilen N.A., Culberson C.R., Field C.O., deLemos A.S., Russo M.W., Schrum L.W. (2016). Processing of miR17-92 Cluster in Hepatic Stellate Cells Promotes Hepatic Fibrogenesis During Alcohol-Induced Injury. Alcohol. Clin. Exp. Res..

[93] Arab J.P., Verma V., Martin-Mateos R., Simonetto D., Kamath P.S., Gores G.J., Shah V., Malhi H. (2018). Extracellular Vesicle C16 Ceramide and S1P Content in Alcoholic Hepatitis Correlates with Disease Severity and Resolution. Gastroenterology.

[94] Eguchi A., Franz N., Kobayashi Y., Iwasa M., Wagner N., Hildebrand F., Takei Y., Marzi I., Relja B. (2019). Circulating Extracellular Vesicles and Their miR “Barcode” Differentiate Alcohol Drinkers With Liver Injury and Those Without Liver Injury in Severe Trauma Patients. Front. Med..

[95] Singal A.K., Shah V.H. (2016). Therapeutic Strategies for the Treatment of Alcoholic Hepatitis. Semin. Liver Dis..

[96] Altamirano J., Miquel R., Katoonizadeh A., Abraldes J.G., Duarte-Rojo A., Louvet A., Augustin S., Mookerjee R.P., Michelena J., Smyrk T.C. (2014). A histologic scoring system for prognosis of patients with alcoholic hepatitis. Gastroenterology.

[97] Arab J.P., Barrera F., Arrese M. (2018). The Evolving Role of Liver Biopsy in Non-alcoholic Fatty Liver Disease. Ann. Hepatol..

[98] Sehrawat T.S., Arab J.P., Liu M., Amrollahi P., Wan M., Fan J., Nakao Y., Pose E., Navarro-Corcuera A., Dasgupta D. (2020). Circulating extracellular vesicles carrying sphingolipid cargo for the diagnosis and dynamic risk profiling of alcoholic hepatitis. Hepatology.

[99] Arab J.P., Sehrawat T.S., Simonetto D.A., Verma V.K., Feng D., Tang T., Dreyer K., Yan X., Daley W.L., Sanyal A. (2020). An Open Label, Dose Escalation Study To Assess The Safety And Efficacy Of IL-22 Agonist F-652 In Patients With Alcoholic Hepatitis. Hepatology.

